# Methods and Research for Multi-Component Cutting Force Sensing Devices and Approaches in Machining

**DOI:** 10.3390/s16111926

**Published:** 2016-11-16

**Authors:** Qiaokang Liang, Dan Zhang, Wanneng Wu, Kunlin Zou

**Affiliations:** 1 College of Electric and Information Technology, Hunan University, Changsha 410082, China; wuwanneng@hnu.edu.cn (W.W.); kunlin@hnu.edu.cn (K.Z.); 2 State Key Laboratory of Advanced Design and Manufacturing for Vehicle Body, Hunan University, Changsha 410082, China; 3 School of Mechanical, Electronic and Control Engineering, Beijing Jiaotong University, Beijing 100044, China; 4 Department of Mechanical Engineering, York University, Toronto, ON M3J 1P3, Canada

**Keywords:** multi-component force sensing system, in-process measurement of cutting force, sensors for unmanned machining

## Abstract

Multi-component cutting force sensing systems in manufacturing processes applied to cutting tools are gradually becoming the most significant monitoring indicator. Their signals have been extensively applied to evaluate the machinability of workpiece materials, predict cutter breakage, estimate cutting tool wear, control machine tool chatter, determine stable machining parameters, and improve surface finish. Robust and effective sensing systems with capability of monitoring the cutting force in machine operations in real time are crucial for realizing the full potential of cutting capabilities of computer numerically controlled (CNC) tools. The main objective of this paper is to present a brief review of the existing achievements in the field of multi-component cutting force sensing systems in modern manufacturing.

## 1. Introduction

Unmanned machining centers such as Computer Numerical Control (CNC) machines are commonly used in manufacturing industries, allowing more accurate and quick fabrication [1,2]. As demands for higher quality, productivity, reliability, and efficiency level of manufacturing processes grow, reliable and effective sensing systems for CNC machines to monitor and inspect the cutting process and the condition of cutting tools have become necessary [3,4,5,6,7,8]. An unmanned machining center equipped with an automated cutting process monitoring system enables higher efficiency of equipment utilization, hence realizes significant manufacturing cost reductions. Among the variety of information provided by the monitoring system, cutting force are essential for achieving the elementary physical mechanics and dynamics of cutting processes.

Force sensing systems have presented themselves in a bewildering variety of industrial automation equipment since the late 1970s [9,10,11,12].

In recent years, cutting force sensing systems have been widely used as the most effective method to identify or monitor the dynamic behavior of cutting processes and the condition of cutting tools. Generally, force sensing systems used in automated machining processes can be divided into the following categories according to their functions:

(a) Characterizing, modeling and optimizing the cutting process. Understanding the metal cutting mechanism such as effects of cutting variables on the cutting force, and then enabling the process planner to decide the optimal cutting conditions such as the cutting speed and feed rate.
(b) Monitoring the cutting tool condition such as cutter deflection and breakage. Predicting the real-time tool wear and tool failure. Analyzing the stresses of the cutter and machine tool. Verifying the conformity of dimensions, surface location error and surface finish to its geometric tolerances.
(c) Detecting the chatter vibrations and stability of the cutting process, providing information useful for mechanical design of cutters, machine tools and their spindle bearings.

In general, the various cutting force sensing systems can be categorized into either indirect or direct methods (Table 1). The indirect sensing approaches detect related parameters that change to some degree with cutting force such as spindle motor current, voltage, acceleration, acoustic emission, and vibration, etc. [13,14,15,16,17,18,19,20,21,22]. On the other hand, the direct sensing approaches are those that employ specific effects caused directly by cutting force. Specifically, these direct effects include mechano-magnetic, mechano-electric, and mechano-optic conversions, etc. [23,24,25,26,27,28,29]. Although the direct measuring approaches are easy to implement and can yield more accurate measurement results, the limitations such as low reliability, high cost, and fragility to overload, etc. prevent such methods from being applied in machine shops [30,31].

As shown in Table 2, the amount of published papers about cutting force sensors per decade reflects a steadily increasing trend in research over the past few decades. In addition, the increase in magnitude of the publishing rate about cutting force sensors represents the rapid development and particular emphasis of the technology.

## 2. Cutting Force Sensing Techniques

Multi-component cutting force sensing system can detect cutting forces along multiple axes by using various measurement techniques or principles. Some commonly studied techniques are based on theoretical models, current sensor, strain gauge, capacitive, optoelectronic, and piezoelectric methods. The selection of a proper technique for cutting force sensing system depends on the advantages and disadvantages of the techniques.

### 2.1. Current-Sensor-Based Cutting Force Sensing Systems

**Figure 1:**
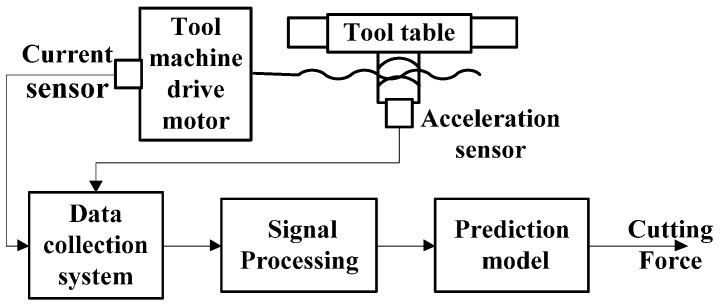
Schematic illustrations of current-sensor-based cutting force sensing technique.

The first current-sensor-based cutting force measurement method was proposed by Taylor through assessing the current of the driving motors of the machine tool in the early years of the 20th century. Since then, all over the world, the number of reported initiatives with the goal of predicting cutting force through current and power measurement have increased dramatically. As shown in the Figure 1, current-sensor-based cutting force sensing approach predicts multi-component force through prediction models such as mathematical, analytical and experimental models with the current signals emitted by the tool machine drive motor [33,34,35,36,37,38,39,40,41].

Shin et al. [14] proposed a method for predicting cutting force in end-milling process with an acceleration sensor and Hall-effect current sensors. Stein et al. [42] proposed a dynamic lumped parameter model to characterize the current of a DC motor of a CNC machine tool, the electric current was used to calculate the cutting force and the forces associated with drive system components. Altintas [43,44] described a model of a vertical milling machine feed drive control system to access the cutting force and predict the tool failures in milling, and the feasibility of the cutting force prediction approach based on armature current measurement was illustrated. Kim et al. [32,45], working with SENA Technologies, Inc. (San Jose, CA, USA), introduced a feasibility study for in-process multi-axis cutting force prediction approach based on an Artificial Neural Network (ANN) system with the input of current signals emitted by the tool machine drive motor, servo motor feed rates. The executed experiments show that the predicted cutting force obtained by the proposed approach agreed with the actual cutting force by comparison with method based on Kalman Filter (KF) disturbance observer. A four-layered ANN (an input layer, two hidden layers, and an output layer) can be used in the experiments. The input data comprised eight components, i.e., the current of two servo motors with one-step behind values, and the federation of two servo motors with one-step behind values. The output layer consists of one node indicating the cutting force. The performance of the proposed approach was influenced by the used training sample obtained under a specific cutting condition. Therefore, the generalization of the ANN needed to be considered further.

Li [46,47] discussed a three-axis cutting force measurement method by using inexpensive Hall-effect current sensors and models of the spindle and feed drive systems in a CNC turning center. An estimation algorithm based on neuro-fuzzy learning algorithm was proposed to estimate the tangential and axial cutting forces, and the axial cutting force was predicted based on the cutting mechanical model. The experimental results showed that proposed approach could measure multi-component cutting forces with an error of less than 25%. Kim and Jeon [48] described an alternative cutting force sensor that measures the motor currents of the feed and spindle system via a 9-channel current sensor in preventing the usage of expensive dynamometers. Additionally, a mathematical model as a function of machining conditions was also formulated to forecast dynamic cutting forces. The maximum root-mean-square error between the measured and predicted cutting force was 25.99 N when the feed rate, spindle speed, and depth of cut were 60 mpm, 1500 rpm and 1.5 mm respectively. The main problem of this approach was that it had limited bandwidths and therefore cannot be used to measure dynamic cutting force.

A novel method to detect cutting force via command voltages drawn by electro-magnetic bearing was described by Auchet et al. [15], and the experimental results show that the calculated cutting forces are in good agreement with a commercial Kistler measurement system. The static accuracy and bandwidth of the proposed measuring system are within 8% and approximately 62 Hz, respectively.

Essentially, the particular connection between the driving motor current and the cutting force of machining process is complicated. For example, the accelerating, decelerating and the friction force in the table and guide way will result in variation of the servo drive motor current. Therefore, it could be difficult to identify the actual relationship via general approaches, especially for machine tools with AC servo motors.

### 2.2. Strain Gauge Cutting Force Sensing Systems

Strain gauge sensing systems, has been widely used as direct measuring approaches to access cutting force in machining processes due to their advantages such as simple construction, high reliability, as well as high and adjustable resolution.

Santochi, Dini and Tantussi [49] described the first integrated strain gauge cutting force sensing system to monitor cutting force in turning processes. The strain gauges were mounted at specific positions on the tool with full bridge arrangement so as to maximize the system sensitivity. An infra-red data communications system was used to send the output of the bridge to the data acquisition system. Experimental results under different conditions demonstrated that the system repeatability and sensitivity were less than ±3% F.S. and 0.24 mV/N, respectively.

Yaldız, and Ünsaçar [30] described a turning dynamometer based on strain gauge and piezo-electric accelerometer to realize static and dynamic measurements of multi-axis cutting forces in turning operations. The proposed dynamometer included an elastic element in four octagonal rings shape, which was bonded with strain gauges that are connected into Wheatstone bridges. Feed force, thrust force and main cutting force are simultaneously detected with a maximum linearity error of 1.4% F.S. and a maximum coupling error of 0.92% F.S. Machining tests using different cutting parameters were performed, and the results indicated that the proposed strain gauge cutting force sensing system was dependable for turning, milling, and drilling operations, etc.

For turning operations, Zhao et al. [50] developed a semi-conductive strain gauge-based cutting force sensing system that was integrated into the cutting tool to measure the two-axis cutting force. A novel elastic element with two mutually perpendicular rings that mounted to the cutting tool and the tool post was designed. This is advantageous to detect the cutting force with high sensitivity and improved natural frequency because it adopted the ingenious elastic element and MEMS fabrication method. The maximum sensitivity and natural frequency of the proposed system are 0.31 mV/N and 771 Hz.

Zhao et al. [51] developed a tri-axial cutting force sensor with high-performance for turning processes. The sensitive element of the sensor was in the shape of two mutual-perpendicular octagonal rings. The static calibration experiment was carried out and the results show that the accuracy and the lowest natural frequency of the proposed sensor are 0.38%–0.83% and 1122 Hz, respectively.

**Figure 2:**
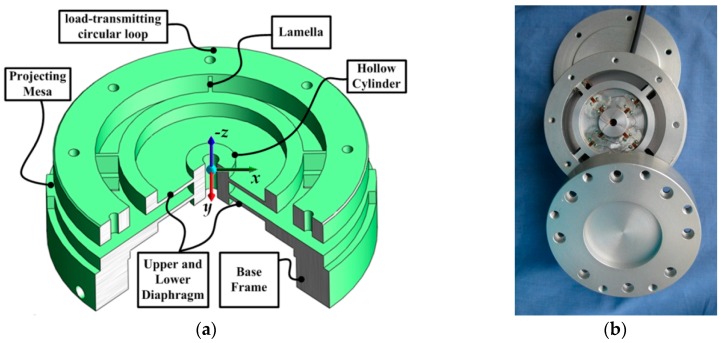
Six-axis strain gauge cutting force sensing system. (**a**) Sketch of the elastic element structure; (**b**) Fabricated prototype of the proposed sensor system.

Recently, our team designed and developed a strain gauge cutting force sensing system [9] to measure six-axis cutting force in machining processes. With a novel elastic element (as shown in Figure 2) and an ingenious arrangement strategy of the mounted strain gauges on that, the sensing system was capable of detecting tangential, normal forces and the moments along three axes simultaneously. Benefitting from a Simulation-Driven Optimization method and an ANN-based decoupling algorithm, the system accessed the multi-axis cutting force with a maximum coupling error and nonlinearity error of 1.47% F.S. and 1.94% F.S., respectively.

Though the strain gauge cutting force sensing systems are capable of providing more accurate measurement, they have limitations like limited bandwidth, high cost, layout constraints, and fragility to overload [52,53,54,55].

### 2.3. Capacitive Cutting Force Sensing Systems

**Figure 3:**
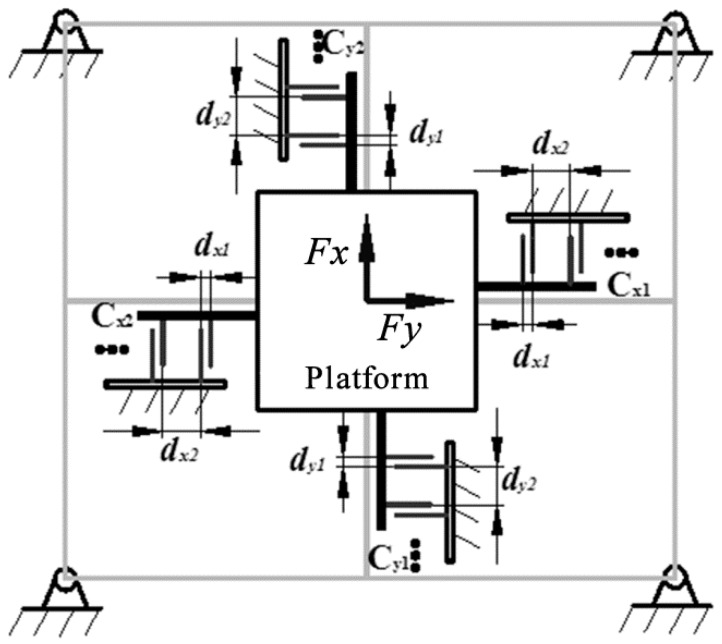
Schematic diagram of a two-axial capacitive force sensor.

Capacitive cutting force sensing systems are one of the most sensitive approaches to access cutting forces and moments with mN even pN resolutions. Estimations show that more than 30% of the modern sensory systems employ capacitive measurement methods. The capacitive cutting force sensing system output can be acquired via switched-capacitor circuits, capacitive ac-bridges, and capacitance to frequency converters. Figure 3 shows a schematic drawing of a two-axial capacitive F/M sensor design. Transverse tri-plate differential comb is used to achieve a high sensitivity and linear input-output relationship [25].

The capacitances of the parallel plate capacitive sensor are given by [25]:

(1) $$C_{x1}=k\frac{\varepsilon_{r}\varepsilon_{0}tl}{d_{x1}}+k\frac{\varepsilon_{r}\varepsilon_{0}tl}{d_{x2}}=\left(1+\frac{1}{n}\right)\frac{k\varepsilon_{r}\varepsilon_{0}tl}{d_{x1}}$$

(2) $$C_{x2}=k\frac{\varepsilon_{r}\varepsilon_{0}tl}{d_{x1}}+k\frac{\varepsilon_{r}\varepsilon_{0}tl}{d_{x2}}=\left(1+\frac{1}{n}\right)\frac{k\varepsilon_{r}\varepsilon_{0}tl}{d_{x1}}$$

(3) $$C_{y1}=k\frac{\varepsilon_{r}\varepsilon_{0}tl}{d_{y1}}+k\frac{\varepsilon_{r}\varepsilon_{0}tl}{d_{y2}}=\left(1+\frac{1}{n}\right)\frac{k\varepsilon_{r}\varepsilon_{0}tl}{d_{y1}}$$

(4) $$C_{y2}=k\frac{\varepsilon_{r}\varepsilon_{0}tl}{d_{y1}}+k\frac{\varepsilon_{r}\varepsilon_{0}tl}{d_{y2}}=\left(1+\frac{1}{n}\right)\frac{k\varepsilon_{r}\varepsilon_{0}tl}{d_{y1}}$$

where *k* is the number of comb finger pairs, *t* × *l* is the common (overlapping) area of the comb fingers, *d*_*x*1_, *d*_*x*2_, and *d*_*y*1_, *d*_*y*2_ represent the initial gap width between electrodes (by initially setting *nd*_*x*1_ = *d*_*x*2_, *nd*_*y*1_ = *d*_*y*2_), *ε*_*r*_ and *ε*_0_ denote the relative and vacuum permittivity, respectively.

For milling operations, Albrecht et al. [56] proposed an indirect cutting force sensing system based on capacitance displacement measurement. A high resolution capacitive sensor (C1-A/B, Lion Precision, Oakdale, MN, USA,) is mounted into the spindle to detect the gap variation between the spindle shaft and the sensor. After calibration with a commercial piezoelectric dynamometer (6255 B, Kistler, Winterthur, Switzerland), the gap variation is proportionally related to the applied cutting force. The static calibration experiment shows the system can measure the cutting force with satisfying linearity performance. However, the limited bandwidth of the system restricted by the structure of the spindle and machine tool is problematic. Benefitting from the proposed Kalman filter scheme based on the frequency response function of the capacitive sensor, the system bandwidth is expanded from 350 Hz to 1000 Hz. For two-dimensional cutting force measurement, Mercedes-star and Cartesian arrangement strategies of capacitive sensors were introduced.

Kim et al. [21] developed a cutting force sensing system for milling operations by detecting spindle displacement with a cylindrical capacitive displacement sensor (CCDS). With the dynamic signals of the spindle tool system obtained from the CCDS, the multi-component dynamic cutting force was predicted. The dynamic cutting force estimation of the system has been shown to work in the laboratory and in the high speed cutting conditions on various machine tools. However, the quantitative analysis of the system performance has not yet been published.

Many of the latest researches and developments associated with cutting force sensing systems involve capacitive sensors that provide precise displacement measurement. Advantages of the systems are their wide frequency bandwidth, high resolution, noncontact measurement, and superior durability. Unfortunately, the capacitive cutting force sensing system suffers from some drawbacks such as complex integration into the machine tools, intrinsic temperature dependence, electrostatic edge effect, etc.

### 2.4. Optoelectronic Cutting Force Sensing Systems

Another common method to detect cutting force in machining relies on optoelectronic sensing. Manufacturing industries with harsh manufacturing environments may benefit from the use of multi-component optoelectronic cutting force sensing systems.

**Figure 4:**
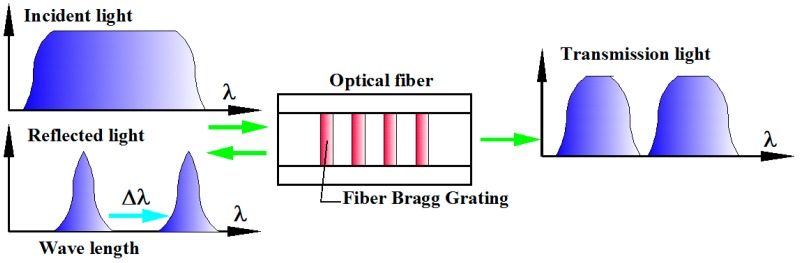
Schematic diagram of optoelectronic cutting force sensing approach based on Fiber Bragg grating sensor.

Fiber Bragg grating sensor, as the most promising optoelectronic force sensing system, have attracted considerable attentions due to their unique opto-electronic properties. Figure 4 shows a schematic drawing of optoelectronic cutting force sensing approach based on Fiber Bragg grating sensor through wavelength division multiplexing techniques.

Liu et al. [31,57] published papers in which they investigated three perpendicular force measurement in milling operations based on Fiber Bragg grating sensor. An elastic body with four rings in annulus shape with pre-tightening device was designed, and the Fiber Bragg grating sensors was fixed on the elastic body. Verification experiments were performed and the results showed that the proposed approach could be adopted in milling and turning operations.

Jin et al. [23] described an approach for measuring cutting force with an optical fiber sensor. The calibration experiment and manufacturing test results demonstrate that the described approach possesses competitive advantage. The natural frequency, sensitivity and linearity of the system are 950 Hz, 2.51 mV/N and 1.2% F.S., respectively.

For dynamic machining applications, Bartow, Calvert, and Bayly [58] proposed an optoelectronic micro machine tool motion sensing system based on Fiber Bragg Grating (FBG) sensors. The FBG sensors were mounted to the cutting tool surface, and precise evaluation of tool tip motion is performed by comparison with a piezoelectric accelerometer. Cutting texts in an actual lathe machining environment were carried out, and the results demonstrated the feasibility of the proposed system.

The competitive advantages of optoelectronic cutting force sensing systems are their wide measurement range, small volume, high reliability, low weight, greater adaptability, and anti-electromagnetic interference. Hence, optoelectronic cutting force sensing systems have attracted significant attention for applications of multi-component cutting force measurement.

### 2.5. Piezoelectric Cutting Force Sensing Systems

**Figure 5:**
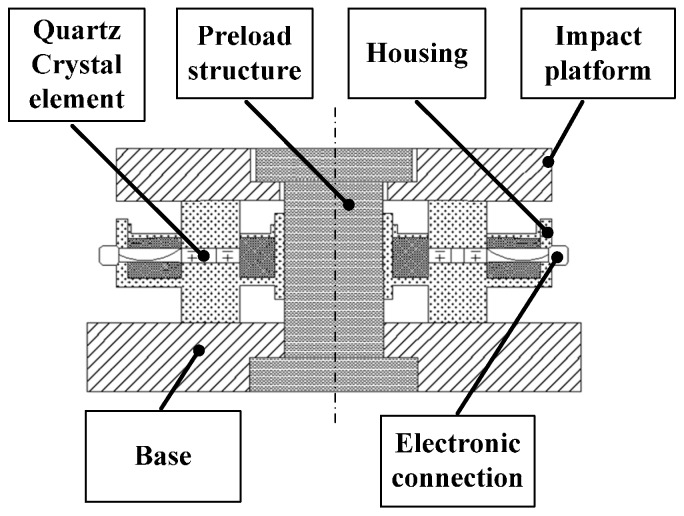
Schematic representation of piezoelectric cutting force sensor.

Most commercial cutting force sensing systems detecting the multi-axis cutting force in milling, turning, drilling or grinding based on piezoelectric method due to its wide dynamic range, high rangeability, good accuracy with finer resolution, high stiffness, smaller size, etc. [59,60,61]. As shown in the Figure 5, the piezoelectric cutting force sensing systems employ the piezoelectric effect of quartz crystal element to detect dynamic changes in cutting force.

Totis et al. [62,63] developed a three-component cutting force measurement based on piezoelectric cutting force sensing technology for turning operation. A commercial tri-axial piezoelectric force sensor (9251A, Kistler, Winterthur, Switzerland) was mounted into the prototype. Static calibration experiments and dynamic response tests of the developed prototype were carried out in a modern CNC lathe. The maximum relative error and static crosstalk disturbances are 6.6% and 3% respectively.

Park et al. [26,64] presented several works on the design and development of cutting force sensing systems to monitor micro- or macro-machining processes. An integrated cutting force sensor system was designed and implemented to detect multi-axis cutting force by integrating piezoelectric force sensors into the spindle. The accuracy and frequency bandwidth of the cutting force sensing system is dramatically increased and that benefits from the proposed compensation method with sensing and the signal processing algorithm. The proposed system is used to optimize the feed rate, and monitor the chatter and tool breakage for milling operation. In another contribution, the authors developed a force sensing system to detect micro-cutting force based on a three-axis piezoelectric force sensor and a commercial accelerometer for micro-machining operations. The accelerometer was used to measure the vibration characteristics, and its signals were employed to increase the accuracy and bandwidth of the obtained multi-axis cutting force.

For on-line inspection of cutting geometry, Rao et al. [20] developed a piezoelectric cutting force sensing systems based on integrated piezoelectric dynamometer (209A02 of PCB Piezotronics Inc., Buffalo, NY, USA) with a resolution of 0.44 mN and a sensitivity of 7 mV/gm. The depth and width of cut is predicted by the proposed theoretical model with a relationship of geometry dimensions and the detected force signal. Experiments results represented that the proposed prediction approach was capable of measuring the cutting geometry with the maximum relative error of 10%.

The piezoelectric cutting force sensing systems suffer from high cost, lag characteristics, mounting constraints, susceptible to the tool and workpiece dynamic characteristics. A further disadvantage of this cutting force measurement method is that the system is inflexible and needs recalibration due to the frequently changing workpieces.

## 3. Application and Processing of the Acquired Cutting Force Information

The prediction of cutting forces in machining is required for supervision of tool wear evolution or failure, access the generated heat, prediction of machined surface and workpiece accuracy, calibration of cutting force coefficients, analysis of material machinabilities, optimization of machining parameters, inspection of metal cutting chip formation, and detecting of chatter [65].

### 3.1. Adaptive Control

How to make a suitable selection of machining process parameters such as the cutting speed, feed rate, cut depth, and rake angles is a recurring problem for the manufacturing industry. Since the variation of cutting force is directly and specifically related to the cutting process conditions, it was realized that the cutting force is one of the most valuable variables to be controlled in the machining process. With the adaptive control of cutting force, the optimal machining parameters such as cutting speed, feed rate of the machining could be chosen. The optimal parameters ensure machining with high chip removal rate as well as prevention of excessive tool wear, breakage.

The need for dynamic detecting and real-time control of cutting force in machining raises various issues, as introduced by Karabulut [66]. Model-based control strategy of cutting force is ineffective due to the inaccurate mathematical model for machining systems. Lian et al. [67] proposed a model-free control strategy based on grey prediction fuzzy control law for constant cutting force in turning operations. To evaluate control performance of the introduced control strategy, an old lathe turning system was converted with an inverter, an encoder, an AC servo motor, a load cell and a compatible PC. The experiment results show that the converted lathe turning system using the grey prediction fuzzy controller is able to achieve the control of constant cutting force with improved performance than using traditional fuzzy controller. A further advantage of this cutting force control method is that the chattering of the cutting tool was significantly suppressed.

Zuperl et al. [68,69] proposed a neural control strategy, cooperating with CNC, to optimize the machining process with constant cutting force in end milling. The control strategy ensures continuous optimization of machining parameters by adaptively adjusting feed rate according to the particular situations. A commercial piezoelectric cutting force sensor (9255, Kistler, Winterthur, Switzerland) was used to simultaneously measure the three-component cutting force. With the introduced control strategy, the cutting force in machining was maintained to be consistent with the reference value, and then the maximum metal removal rate was obtained to significantly reduce manufacturing cost and machining time by approximately 24%. The stability and robustness of the proposed feed-forward neural control scheme (FFNCS) were confirmed by the simulation and machining experiments.

Despite growing interest from both research institutes and commercial companies in designing and developing reliable, robust cutting force control strategy for manufacturing progresses in different cutting conditions, the utilization of these strategies in commercial CNC machine tool control systems has been minimal. However, modern machine tools with open architecture controllers are the key enabler for the progressive realization of adaptive cutting force control to increase manufacturing efficiency, avoid excessive cutting tool wear, and prevent potential tool breakage.

### 3.2. Predicting and Evaluating Real-Time Tool Wear and Tool Breakage

Multi-component cutting force sensing systems have gradually become known as the most reliable indicators of important cutting action information, and have been widely used to predict and evaluate cutting tool conditions such as wear and breakage of the cutting tool [70,71,72,73,74].

Intelligent or reliable evaluation and prediction of cutting tool wear condition based on cutting force sensing system is capable of preserving the cutting tools, CNC machine tools servo drive systems, and manufacture components in metal cutting operations. Li et al. [47] proposed an intelligent monitoring system for cutting tool wear condition based on a fuzzy classification method and a current-sensor-based cutting force sensing system. Cutting force in turning processes was obtained via a neuro-fuzzy approach with the feed rate and feed motor current. Cutting tool wear condition was estimated with the fuzzy classification method. The experimental results demonstrated that the accurate prediction of the cutting tool could be achieved over a range of cutting conditions. A further advantage of this estimation approach is that the system is inexpensive due to the current-sensor-based cutting force measurement approach. Additionally, a number of investigators have used current-sensor-based cutting force estimation approach for the analysis of cutting tool wear in different cutting operations [75,76,77].

Balsamo et al. [78] proposed a multi-sensor monitoring system to identify Catastrophic Tool Failure (CTF). The system consists of a commercial tri-axial force sensor (9017B, Kistler, Winterthur, Switzerland) mounted on the clamps and an acoustic emission sensor (8152B, Kistler, Winterthur, Switzerland). A reliable detection methodology for cutting tool breakage based on the combined information from the sensors was developed for turning operation.

For machining operations with some stainless steel materials of hard machinability, evaluation of the cutting tool wear becomes even more significant and complicated. Selvaraj et al. [79] analyzed the influence of machining cutting speed and feed rate on cutting force, surface roughness of manufactured part, and cutting tool wear in turning progress of nitrogen alloyed duplex stainless steel. They disclosed that cutting force and surface roughness were strongly affected by the feed rate parameter, while the cutting tool wear condition was primarily affected by the cutting speed. The Taguchi optimization methodology was used to optimize cutting parameters in turning progresses when machining duplex stainless steel.

In a similar effort, Rizal et al. [80] estimated cutting force by multi-component strain gauge sensing systems integrated on the cutting tool holder, and proposed a cutting tool wear monitoring approach based on I-kaz 2D and I-kaz™ methods. The relationship between the cutting force signal and the progression of cutting tool wear has been derived from the turning machining texts. It has been found that cutting force shows an increasing rate, following the rising of flank wear evolution. In addition, the feed cutting force increased more massively than the main cutting force due to the progression of the cutting tool flank wear.

For tool wear detection and fault diagnosis in metal cutting processes, Huang et al. [81] developed an inexpensive tool fault detection and diagnosis approach based on an observer model. A commercial cutting force dynamometer was mounted into the machining center to evaluate tool wear, and a camera system was used to estimate the amount of tool wear. Several cutting processes under different cutting conditions were carried out and the results showed that the proposed approach was effective.

Multi-component cutting force has a great impact on cutting tool wear and failures. Consequently, multi-component cutting force sensing systems are emphasized as critical component in the modern manufacturing systems due to technical and economic reasons. To minimize the excessive tool wear and improve the tool life, different sensing systems and monitoring methods based on Artificial Intelligence (AI) can be utilized for monitoring and controlling the cutting tool wear condition to prevent additional costs. Among the various sensing and monitoring systems, cutting force sensing systems are the most efficient. However, the system performance still needs to be improved to be applied into the actual automatic manufacturing systems.

### 3.3. Detecting and Controlling Chatter

Cutting tool chatter, also called self-excited cutting tool vibrations, results in negative effects such as rough surface finish, poor workpiece accuracy, unwanted noise, reduced product quality and industry productivity. Generally, chatter in metal cutting operations originates from the dynamic interaction effect between structural and cutting dynamics [82,83]. Among the numerous detection and control strategies for chatter in automatic manufacturing systems introduced in the literature, methods based on dynamic cutting force re drawing more and more attention for their intrinsic relationship with dynamic systems.

Tlusty et al. were among the first to propose studying machine tool chatter in 1958 [84,85]. Since then, a number of research teams have carried out work on detecting and controlling chatter to improve the quality and productivity in the manufacturing industry [86,87,88,89].

For the cutting chatter stability lobes prediction method, Peng et al. [90] proposed a novel prediction approach based on simulation model of cutting force and support vector machine (SVM) theory. Multi-component cutting force along *x*- and *y*-axis was detected with a commercial cutting force sensing system (9257 B, Kistler, Winterthur, Switzerland) and used to recognize cutting chatter. Feature extraction of multi-component cutting force signals was performed based on wavelet energy entropy theory. Experimental tests carried out on a CNC machining center, and the results demonstrate that the prediction based on SVM can identify the cutting chatter with an accuracy rate of 98.333%.

The traditional manufacturing industry always employs conservative cutting conditions to prevent cutting chatter, which results in low productivity and high cost. Dynamic cutting force was widely estimated to predict the cutting chatter reliably and accurately. However, very few common or industrial chatter detection and control systems based on multi-component cutting force measurement are available. In addition, methods for detecting and controlling cutting chatter always need to be amended according to the specific cutting tool, workpiece, tool path, cutting conditions, machine tools, etc.

### 3.4. Other Applications with Multi-Component Cutting Force Information

In recent years, emerging areas of modern manufacturing industry have resulted from the application of multi-component cutting force information [91]. For example, Risbood et al. [92] proposed a prediction method based on neural networks for surface finish and dimensional deviation with multi-component cutting force and vibration sensing system. Ramesh et al. [93] analyzed error sources induced by cutting-force associated with geometric and fixture dependent errors. Accordingly, error compensation or elimination methods for every kind of error were proposed. Segreto et al. [94] proposed a multiple sensor monitoring system that consists of a cutting force sensor and an acceleration sensor to estimate the Ni-Ti alloy workpiece machinability in turning operation. The correlation between the pattern feature vectors from the sensorial data and the process quality was investigated with a pattern recognition method based on an adaptive Neuro-fuzzy inference system. The experimental results showed that the proposed method significantly decreased the average Root Mean Square Error (RMSE) to 0.012. Due to the advantages of multi-component cutting force sensing systems, they have been shown to be suitable in more various applications.

## 4. Discussion and Conclusions

Regardless of the fact that much research and development has been carried out in recent decades, significant work is urgently needed before accurate, reliable, and cost effective multi-component cutting force sensing systems will be commonly adopted in modern machine tools. Over the past decade, our team has designed and developed various multi-component force sensing systems aiming at force measurement in robotic manipulation and unmanned machine operations. For the future directions and challenges of multi-component cutting force sensing system, the following conclusions and observations can be reached based on our review:

- Multi-component cutting force sensing techniques: the emergency of new force sensing technology such as vision-based and fiber Bragg grating sensors-based sensing techniques, MEMS-based sensing systems, optoelectronic based devices, and film based sensors will make it possible to measure the multi-component cutting force with higher performance. Specifically, piezoelectric cutting force sensing system is ideally suited for the detection of the multi-component cutting force due to its high frequency response and high dynamic range. Other sensing techniques can be used in different machining applications. For example, vision-based force sensing technology for micro-machining operations.
- Frequency bandwidth: the strain gauge cutting force sensing system is restricted to be applied in the high speed machining operations due to its limited frequency bandwidth. Dynamic behavior of the machining system is various with the machine tool type, cutting conditions, workpiece and cutting tools. Therefore, multi-component cutting force sensing system with superior dynamic performance can achieve stable long-term frequency behavior, and will be used as a common sensing system for different machining operations.
- From single component to multiple components: more and more cutting force sensing systems for modern machine tools can measure the force with multiple components. Right now the commonly available systems always have two or three force components, and it will be remarkable to upgrade the sensing system with three additional torque components.
- Overload protection: to avoid fatal damage due to excessive cutting force, overload protection in both mechanism and software keeps drawing attention in the design and development of multi-component cutting force sensing systems. Additionally, overload protection is capable of preventing accuracy variation of the machining operations under excessive cutting conditions. However, no mature overload protection for multi-component cutting force sensing system or unmanned machining operations is available until now.
- Stiffer cutting force sensing systems: the stiffness of the machining system depends on the component with minimum stiffness. For direct cutting force measurement approaches, the cutting force sensing system is always the weakest component in the force-flux flow of the machining system. Therefore, the modern machine tools can benefit from cutting force sensing system with higher stiffness. Simulation-driven design, multi-objectives optimization, and optimization exploration could be used to design and develop stiffer cutting force sensing systems.
- Multi-sensor data fusion system: other than cutting force sensing systems, other kinds of sensors such as accelerometers, dimensional, proximity, vibration and acoustic emission sensors and their signals could be used to gather more valuable and accurate information. Feature extraction processes and decision making methods are helpful to obtain useful features and improve the efficiency of the inspection.
- Modern machine tools and smart machining: intelligence level of the manufacturing process could be heightened by using adaptive cutting force control, on-line monitoring real-time tool wear and tool breakage, error compensation, etc.

## Figures and Tables

**Table 1:** Intrinsic transduction techniques for cutting force measurement [9].

Measurement Type	Sensing Principle	Macroscopic Description	Advantages	Disadvantages	Typical Development
Indirect approach	Current	Current consumption of the driving motors of the machine tool	easier to achieve cost effective	time-consuming unsuitable for multi-axis cutting process without consideration of the frictional behavior of the machine tools	[32]
	Voltage	Command voltages of magnetic bearings	wide bandwidth (up to 4 kHz) easy to conversion and processing	Limited to stable conditions susceptible to electromagnetic interference requires magnetic bearing presence	[15]
Direct approach	Strain gauge	Change in resistance due to cutting force	simple construction high and adjustable resolution high reliability	higher power consumption rigid and fragile scarce reproducibility contradictions between flexibility and sensitivity narrow frequency bandwidth	[22]
	Capacitive	Change in capacitance due to cutting force	high sensitivity and resolution long-time stability Adaptability to Environment	temperature sensitive stray capacitance Edge effect	[21]
	optoelectronic	Change in refractive index due to cutting force	good reliability wide measurement range good adaptability to environment	non-conformable hard to construct dense arrays	[23]
	Piezoelectric	Generation of surface charge due to cutting force	high frequency response and high dynamic range higher accuracy and finer resolution high sensitivity and stiffness	charge leakages poor spatial resolution deteriorations of voltages or drifts in the presence of static forces require to be embedded into the machining structure	[20]

**Table 2:** Count of published papers about cutting force sensors from 1970.

Year	IEEE Library	ASME Digital Conllection	Compendex	Springer-Link	SPIE Digital Library
1970–1979	8	0	8	22	2
1980–1989	19	2	67	60	15
1990–1999	94	17	185	129	76
2000–2009	227	442	436	258	429
2010-present	248	677	523	522	745
